# Peripheral Markers of Depression

**DOI:** 10.3390/jcm9123793

**Published:** 2020-11-24

**Authors:** Aleksander Nobis, Daniel Zalewski, Napoleon Waszkiewicz

**Affiliations:** Department of Psychiatry, Medical University of Bialystok, pl. Brodowicza 1, 16-070 Choroszcz, Poland; daniel.zalewski@umb.edu.pl (D.Z.); psych@umb.edu.pl (N.W.)

**Keywords:** depression, biomarkers, inflammatory, interleukins, oxidative stress, brain-derived neurotrophic factor (BDNF), panels, melancholic, atypical

## Abstract

Major Depressive Disorder (MDD) is a leading cause of disability worldwide, creating a high medical and socioeconomic burden. There is a growing interest in the biological underpinnings of depression, which are reflected by altered levels of biological markers. Among others, enhanced inflammation has been reported in MDD, as reflected by increased concentrations of inflammatory markers—C-reactive protein, interleukin-6, tumor necrosis factor-α and soluble interleukin-2 receptor. Oxidative and nitrosative stress also plays a role in the pathophysiology of MDD. Notably, increased levels of lipid peroxidation markers are characteristic of MDD. Dysregulation of the stress axis, along with increased cortisol levels, have also been reported in MDD. Alterations in growth factors, with a significant decrease in brain-derived neurotrophic factor and an increase in fibroblast growth factor-2 and insulin-like growth factor-1 concentrations have also been found in MDD. Finally, kynurenine metabolites, increased glutamate and decreased total cholesterol also hold promise as reliable biomarkers for MDD. Research in the field of MDD biomarkers is hindered by insufficient understanding of MDD etiopathogenesis, substantial heterogeneity of the disorder, common co-morbidities and low specificity of biomarkers. The construction of biomarker panels and their evaluation with use of new technologies may have the potential to overcome the above mentioned obstacles.

## 1. Introduction

Depression, or Major Depressive Disorder (MDD) is the most prevalent psychiatric disorder worldwide and a leading cause of disease burden [1]. It is mainly characterized by depressed mood, anhedonia, sleep and appetite disturbances, loss of interest or pleasure in activities once enjoyed and feelings of guilt or worthlessness. A high suicide rate among individuals suffering from the disorder is the darkest side of depression. Currently affecting around 300 million people worldwide and with 5%–17% of the population suffering from the disorder at least once in their lifetime, depression is a major clinical, emotional and socioeconomic burden for society. The World Health Organization (WHO, Geneva, Switzerland) estimates that, by 2030, depression will have become the leading cause of disability worldwide [2]. An important issue in depression is that of low remission rates. Only approximately half of the patients achieve complete remission [3] and with each subsequent treatment remission rates decrease [4]. The efficacy of classical antidepressant drugs, which target the monoamine systems, is only marginally higher than that of a placebo [5]. An urgent need exists to find biomarkers in order to better understand the pathogenesis of depression, monitor treatment outcomes and identify new drug targets [6]. The aim of the study was to provide a comprehensive review of potential depression markers. For some, currently available evidence is insufficient to allow for regarding of them as biomarkers sensu stricto. However, alterations in their concentrations may provide relevant information concerning the pathophysiology of depression and be a starting point for future, larger biomarker studies.

### 1.1. Concepts of Depression

The etiopathogenesis of depression is highly complex and therefore still not fully understood. Although the monoaminergic theory of depression is now universally accepted, other pathologies have also been found to be associated with the development of the condition. Research to date has focused mainly on stress axis dysregulation (hypothalamus–pituitary–adrenal, HPA), oxidative stress-induced damage [7,8,9,10], hippocampal and frontal lobes dysfunction (neurodegeneration) [11,12], and neurotoxic, inflammatory and immunological processes [7,13,14,15,16,17]. In recent years, knowledge of genetic and epigenetic factors which could contribute to depression has expanded. Furthermore, many psychological hypotheses seek to explain the causes of depression (e.g., learned helplessness hypothesis) [18]. Finally, the neurodevelopmental theory of depression attempts to combine previous approaches with particular emphasis on the impact of the earliest stages of a person’s life on MDD occurrence [19].

### 1.2. Heterogeneity of Depression

Depression is a heterogeneous disorder. To date, no universally accepted classification of depression subtypes has been developed. Most authors acknowledge the existence of melancholic and atypical subtypes [20], but others also mention the following subtypes: dopamine-related subset with anhedonia, inflammatory subset [21], the existence of which is questioned by others [22], suicidal depression [23], anxious depression, depression with functional and somatic traits (closely related to chronic fatigue syndrome) [24], reactive depression, psychotic depression, dysthymia [11], depression with panic attacks, depression in people with obsessive traits, depression accompanying a known physical illness, and pseudo-demented depression [25]. According to some authors, MDD should not be regarded as a single disease but rather a group of diseases with distinct causes, patho-physiologies and symptomatologies [11,26].

### 1.3. Diagnosis of Depression

The diagnosis of depression is currently symptom-based. There are several psychometric scales which help clinicians to assess different dimensions of depressive functioning, of which the most commonly used are the Hamilton Depression Rating Scale (HDRS) [27], Montgomery-Asberg Depression Rating Scale (MADRS) [28] and Beck Depression Inventory [29]. The International Classification of Diseases (ICD) (from the 6th to the 11th edition) and the Diagnostic and Statistical Manual (DSM) (from I to V edition) provide a set of criteria for diagnosing depression. However, they are based on the patient’s self-reports and the clinician’s observations of patient behaviour [30]. Neither DSM nor ICD makes reference to any objective, measurable biological features that could assist in diagnosing depression. This makes the entire diagnostic process subjective, to a certain degree, and leads to a considerable risk of misdiagnosis and suboptimal treatment, which may last for many years. By way of illustration, bipolar disorder (BPD) in its depressive phase is frequently misdiagnosed as MDD [31]. Furthermore, depressive symptomatology cannot be clearly understood and properly codified in psychiatric classifications without a thorough knowledge of the neurobiological, pharmacological and etiological mechanisms underlying the development of depression [9]. Another important issue is the contradictory character of several symptoms of depression in DSM (e.g., increased or decreased body mass or appetite, hyper- or hyposomnia, decreased or increased activity). A few distinct or even opposite clinical pictures can be built based on these criteria. Hence, the use of ‘depression’ as an umbrella term could lack biological validity [32,33]. Additionally, the discovery of objective, biological markers of depression would not only be an invaluable help for clinicians but could also serve as a springboard for improving our understanding of the biology of depression with its various subtypes.

### 1.4. Biomarker Subtypes

**Figure 1:**
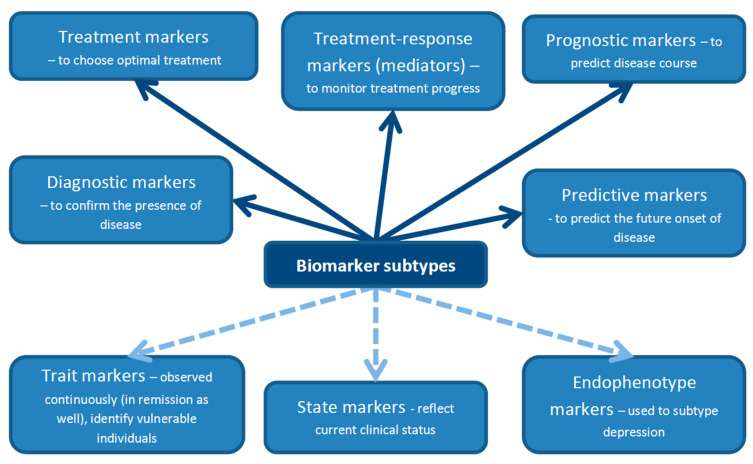
Biomarkers can be divided into several subtypes according to their function (top half of the figure, solid lines) or based on when they can be observed (bottom half of the figure, dashed lines).

A biomarker is defined as a characteristic that can be objectively measured and evaluated as an indicator of physiological processes, pathogenic processes or responses to a therapeutic intervention [34]. Markers should not be confounded with characteristics of a particular disease [35]. There are several classifications of biomarkers described in the literature. For the purposes of this review, we adopted (with modifications) a classification proposed by Lopresti et al. [23] presented in Figure 1. Biomarkers can be divided into diagnostic biomarkers, which are used to confirm the presence or absence of disease; treatment biomarkers, which could be helpful in selecting optimal treatment for a particular patient from a range of available therapeutic options; treatment–response biomarkers (also called mediators) to measure treatment progress; prognostic biomarkers to predict disease course; and, finally, predictive biomarkers, whose role is to predict the future onset of disease [23]. Biomarkers can also be classified as trait, state and endophenotype biomarkers [36]. Trait biomarkers are those which can be observed continuously—not only in the acute phase of the disease, but also in remission or even before disease onset. The last characteristic makes them similar, to a certain degree, to predictive biomarkers. Trait biomarkers may help identify vulnerable individuals. State biomarkers reflect the current clinical status of the patient. They are present during the acute phase of the disease or shortly before disease onset, but they disappear in remission. Endophenotype biomarkers are useful in subtyping depression. They are based on the relationship between depressive phenotypes and specific genetic factors [36].

Biomarkers should be highly sensitive and disease-specific (>80%) to be clinically useful [37]. Receiver operating characteristic area under the curve (ROC AUC) is a measure of biomarker accuracy, with ROC AUC < 0.5 suggesting poor accuracy, and ROC AUC close to 1 suggesting high accuracy [38].

### 1.5. Biological Systems Affected in Depression

**Figure 2:**
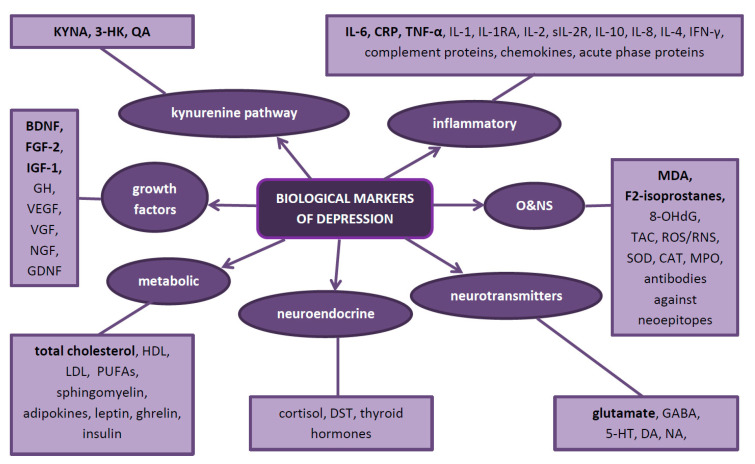
The summary of the most important depression markers. **In bold**—those confirmed by a recent umbrella meta-analysis [40]. Abbreviations: 3-HK—3-hydroxykynurenine; 5-HT—serotonin; 8-OHdG—8-hydroxy-2-deoxiguanosine; BDNF—brain-derived neurotrophic factor; CAT—catalase; CRP—C-reactive protein; DA—dopamine; DST—dexamethasone suppression test; FGF-2—fibroblast growth factor-2; GABA—gamma-aminobutyric acid; GDNF—glial cell line derived neurotrophic factor; GH—growth hormone; HDL—high-density lipoprotein; IGF-1—insulin-like growth factor-1; IL-1—interleukin-1; IL-2—interleukin-2; IL-4—interleukin-4; IL-6—interleukin-6; IL-8—interleukin-8; IL-10—interleukin-10; IL-1RA—interleukin-1 receptor antagonist; INF-γ—interferon-γ; KYNA—kynurenic acid; LDL—low-density lipoprotein; MDA—malonylo-dialdehyde; MPO—myeloperoxidase; NA—noradrenaline; NGF—nerve growth factor (NGF); O&NS—oxidative and nitrosative stress; PUFAs—polyunsaturated fatty acids; QA—quinolinic acid; ROS/RNS—reactive oxygen/nitrogen species; sIL-2R—soluble interleukin-2 receptor; SOD—superoxide dismutase; TAC—total antioxidant capacity; TNF-α—tumor necrosis factor-α; VEGF—vascular endothelial growth factor; VGF—VGF nerve growth factor.

**Figure 3:**
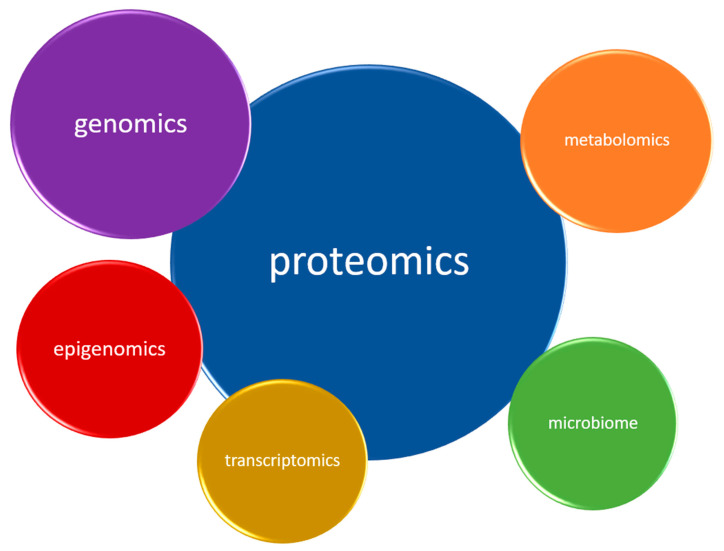
Biological systems involved in depression can be assessed on different measurement ‘levels’, called ‘omics’. Note that every system can theoretically be assessed on each ‘omic’ level.

In depression, as evidence to date suggests, five biological systems are mainly affected. Therefore, they constitute natural sources of potential biomarkers. These are the inflammatory, neurotransmitter, neuroendocrine, neurotrophic and metabolic systems (Figure 2) Each system can be assessed at different biological levels (this is called the ‘omics’ approach)—from genomic and epigenomic, through transcriptomic and proteomic to metabolomic (Figure 3). It is worth emphasizing that not every technique is equally efficient in the evaluation of a particular system. Apart from the ‘omics’ mentioned above, there is a growing understanding of the human microbiome and its impact on mental health. Therefore, its assessment might help establish a more accurate diagnosis and provide more appropriate treatment for depression [39].

## 2. Methods

A literature search was conducted in PubMed, Scopus and Web of Science databases using keywords: ‘depression’, ‘biomarker’, ‘marker’, ‘proteomic’, ‘metabolomic’, ‘inflammatory’, ‘growth factors’, ‘cytokines’, ‘kynurenine’, ‘oxidative stress’, ‘genetic’, ‘subtypes’, ‘melancholic’, ‘biosignature’ as well as combinations of these terms. Relevant articles were then included with the intention to cover the widest possible spectrum of different markers for depression.

## 3. Inflammatory Findings in Depression

The link between inflammation and depression has been conclusively proven and widely reviewed [13,41,42]. In approximately 30% of patients with MDD, the inflammatory response is disturbed [43] and patients with inflammatory disorders have higher depression rates. The distribution of inflammatory markers elevation in a depressive population is continuous and does not allow for distinguishing a distinct inflammatory subtype of depression [22].

Chronic, low grade inflammation is the way through which behavioral and social variables impact health [44]. Depression is associated with chronic low-grade inflammation and is compared to a chronic cold [15]. Patients suffering from autoimmune and atopic disorders, metabolic syndrome, obesity, tobacco dependence, dental caries, atherosclerosis—all of which are associated with increased inflammation—are at a higher risk of depression (Berk et al., 2013). Inflammation is described as a major mediator in the development of MDD and the metabolic syndrome [45]. However, mild chronic inflammation is only a general concept which does not adequately explain the pathophysiology of depression [46].

While it is unlikely that depression is simply an inflammatory disorder, enhanced activation of the immune system in individuals with depression is not merely coincidental. There is substantial evidence confirming the involvement of inflammatory factors in the pathogenesis of depression [47,48,49,50,51,52]. Inflammation is present not only during depressive episodes—elevated levels of inflammatory factors significantly contribute to the occurrence of the first depressive episode [53,54,55]. Physiological and psychological stress, the most important risk factor for depression, has been proven to cause an immune challenge for the body and provoke an inflammatory response [56,57]. Smoking and obesity are common in depression [58,59] and can influence the concentration of inflammatory markers [60,61]. Continuous elevation of cytokines leads directly to increased levels of cortisol by stimulating the HPA axis and increasing corticotropin-releasing hormone (CRH) production [48] and, indirectly, by inducing glucocorticoid resistance, to neurotransmitter concentration changes which are interpreted by the brain as stressors [62,63]. Additionally, elevated cytokine levels cause an increase in the expression of serotonin transporter and induce indoleamine 2,3-dioxygenase (IDO) activity, thus enhancing the kynurenine pathway in the brain. All these factors contribute to the development of depression [64]. Increased inflammation leads to cognitive decline and is likely to be responsible for the impairment of cognitive function observed in depression [65].

The pro-inflammatory state, reflected by elevated levels of pro-inflammatory cytokines, manifests itself externally in what is termed ‘sickness behaviour’, characterized by anhedonia, weight loss, anorexia, memory dysfunction and impaired social interaction—symptoms that occur in MDD [66]. Central or peripheral administration of pro-inflammatory cytokines, such as interleukin-1 (IL-1), interleukin-2 (IL-2), interleukin-6 (IL-6), interleukin-8 (IL-8), interferon-γ (INF-γ) or tumor necrosis factor-α (TNF-α), may produce sickness behaviour [67,68,69,70,71].

The presence of inflammation, however, is not specific to MDD and could be an indicator of two other major psychiatric disorders: schizophrenia and BPD, indicating the possibility of common underlying pathogenetic pathways in disorders involved in immune dysfunction [72,73]. Dubois et al. [72] found that levels of inflammatory biomarkers are similarly elevated in all three disorders and are more closely related to their stage and severity than to a particular disease. Variations in cytokine concentrations could also predict the risk of disease occurrence and treatment resistance in the above mentioned conditions.

Danese et al. [73,74] revealed that people who were subjected to maltreatment or abuse in their childhood were more prone to developing depression in adult life, which may be associated with a predisposition to prolonged stress reactions (HPA axis dysregulation) and stronger inflammatory responses in these individuals. Interestingly, a similar effect—a higher risk of depression—was observed in adults who suffered from a serious illness in their childhood [75].

Since 1980s, with new discoveries emerging in the field of biological psychiatry, there has been a controversy over whether depression is characterized by immune activation or immune suppression. Both hypotheses were supported by convincing evidence. The lowered lymphocyte transformation test (LTT) [76] and blunted natural killer cell activity (NKCA) [77] in depressive patients indicate immunosuppression. At the same time, a large body of evidence supported the role of immune activation in depression—enhanced levels of pro-inflammatory cytokines and acute phase proteins were observed, together with immune cells activation [7,13,14,15,16,17].

To cut the Gordian knot, Maes and Carvalho [78] proposed a concept of two opposite systems which act simultaneously in depression and counter-regulate each other: the immune-inflammatory response system (IRS) and the compensatory immune-regulatory reflex system (CIRS). Both of them are closely interrelated—the activation of IRS inevitably entails the activation of CIRS and the activated CIRS opposes the inflammatory action of IRS. This interplay ultimately leads to the extinction of the inflammatory response, which could be responsible for the self-limiting character of depression. However, following the first depressive episode, once IRS and CIRS have been activated for the first time, there is no return to the homeostatic status quo, and subsequent episodes are characterized by a sensitized immune response, which could explain why successive depressive episodes frequently occur without a tangible cause and are more severe. Greater severity of MDD has been linked to increased activation of both IRS (neopterin, sIL-6R) and CIRS (sTNF-α, sIL-1RA, IL-10) [78]. The pro-inflammatory cytokine IL-6 (IRS) enhances the production of anti-inflammatory sIL-1RA, IL-10 and glucocorticoids (CIRS) [79,80]. Haptoglobin—an acute phase protein—has anti-inflammatory effects and acts as an antioxidant [81]. An increase in IL-1β in depression (M1 activation) is accompanied by an increase in soluble IL-1 receptor antagonist (sIL-1RA) (reflex inhibition). A precise description of IRS and CIRS is beyond the scope of this review and can be found in a publication by Maes and Carvalho [78]. The main conclusions from this paper are presented in Table 1 [78].

Many antidepressant drugs have anti-inflammatory properties which could partly explain their efficacy in the treatment of depression [82]. Antidepressants reduce the production of pro-inflammatory cytokines and increase concentrations of anti-inflammatory cytokines. In a recent meta-analysis, antidepressant treatment was found to attenuate IL-1Β, IL-6 and IL-10 levels as well as M1 polarization of macrophages [83]. Interestingly, an inverse relationship was also observed: anti-inflammatory drugs such as celecoxib, ibuprofen or TNF-α blocker infliximab were described as having antidepressant properties [84,85,86].

Among inflammatory markers, IL-6, C-reactive protein (CRP), TNF-α and soluble interleukin-2 receptor (sIL-2R) appear to have the greatest potential to serve as markers for depression.

### 3.1. Interleukin-6

#### 3.1.1. As a Diagnostic Biomarker

Among all inflammatory cytokines, an increased concentration of IL-6 is probably the most widely and consistently reported in depression. This relationship has been confirmed by several meta-analyses [40,42,47,53,83,87,88,89,90]. Increased IL-6 could be an early marker for cognitive decline in depression. It also corresponds to depression severity and increased HPA axis activity [91,92,93]. Kunugi et al. [21] proposed the use of cerebro-spinal fluid (CSF) IL-6 levels as a bio-marker for the neuro-inflammatory subtype of MDD.

#### 3.1.2. As a Treatment–Response Biomarker

Apart from being a potential diagnostic (state) biomarker, IL-6 has potential to serve as a treatment-response biomarker. Levels of IL-6 decrease along with successful antidepressant treatment [83], which could suggest that antidepressants have anti-inflammatory properties. Decreased levels of leukocytic mRNA for IL-6 have been correlated with effective treatment [94] while persistently elevated serum IL-6 concentrations seem to be characteristic of treatment-resistant depression (TRD) [79]. Interestingly, electroconvulsive therapy (ECT) is reported to increase IL-6 levels [95]. In a meta-analysis by Hiles et al. [88], higher baseline IL-6 levels were correlated with a more significant decrease in depressive symptoms following antidepressant treatment. Furthermore, a meta-analysis by Strawbridge et al. [96] demonstrated that IL-6 levels decrease along with antidepressant treatment administration, regardless of the outcome.

#### 3.1.3. IL-6 Trans-Signaling

IL-6 can exert its biological activity via two separate signaling pathways—the classical pathway and trans-signaling. In the classical pathway, IL-6 binds to membrane-bound IL-6 receptor (IL-6R), present on a few cell types in the body. Elevated levels of pro-inflammatory cytokines may cause cleavage of IL-6R from the cell surface into the bloodstream. The activation of soluble IL-6 receptors (sIL-6R) is responsible for IL-6 trans-signaling [44], which is characteristic of inflammation, while IL-6 classic signaling contributes to anti-inflammatory effects [97]. To assess the impact of IL-6 on the body, it is crucial to measure both IL-6 and sIL-6R concentrations. Elevated sIL-6R levels combined with higher IL-6 concentrations indicate enhanced IL-6 trans-signaling, and thus enhanced inflammation [83,98,99,100]. A study by Maes et al. reported elevated sIL-6R levels in depression [80]. Further studies specified that enhanced IL-6 trans-signaling is characteristic of an acute (current) depressive episode (melancholic or atypical) compared to a remitted state and is a distinctive feature of TRD and melancholia [80,99,100].

### 3.2. C-Reactive Protein

#### 3.2.1. As a Diagnostic Biomarker

CRP is the most frequently used marker of inflammation. CRP is produced in the liver and its secretion is stimulated by IL-6 [44]. The majority of studies have demonstrated that CRP levels are elevated in depression [22,40,42,47,89,96], although no causal relationship has been established between an enhanced CRP concentration and depression [101]. A subgroup analysis revealed that higher CRP levels may be characteristic of depressed men [102,103,104], atypical depression [105], depression with somatic symptoms [106], depressed patients with a history of childhood trauma [107] and cumulative depressive episodes [108]. Elevated CRP levels have also been suggested to be more specific for female than for male patients with MDD [109]. However, it should be noted that enhanced CRP levels are not specific to depression—they are also present in euthymic BPD and manic episodes [110].

#### 3.2.2. As a Predictive Biomarker

CRP also shows potential as a predictive biomarker. Pasco et al. [54] reported that high sensitivity CRP (hsCRP)—a more sensitive measure of inflammation—is an independent risk factor of depression and its elevated levels can predict de novo MDD occurrence. These findings were confirmed in a meta-analysis by Valkanova [89]. Higher CRP concentrations have been linked to an increased risk of hospitalization due to depression [111].

#### 3.2.3. As a Prognostic Biomarker

In the majority of studies, low baseline CRP levels were found to be associated with a better and faster response to selective serotonin reuptake inhibitor (SSRI) treatment [109,112,113]. However, the results are inconsistent. According to some studies, higher baseline CRP levels could predict a better response to treatment [88,114], whereas in other studies they are reported to have no impact on treatment results [115].

#### 3.2.4. As a Treatment Biomarker

Altered CRP levels may help clinicians select an optimal treatment strategy for a particular patient since high baseline CRP levels predict a better response to pharmacotherapy in comparison to psychological therapy [116]. When it comes to drug selection, altered CRP levels might predict the outcome of treatment with escitalopram and nortriptyline (with opposite effects) [112]. Antidepressant treatment reduces CRP concentration marginally [88].

The production of CRP in the liver is induced by TNF-α. Depressive patients with higher baseline CRP levels show a more significant improvement following the injection of an anti-TNF-α drug, infliximab [117].

Most studies investigating inflammatory markers of depression have focused on CRP and IL-6. This approach, however, is too narrow and does not address the complexity of immune-inflammatory processes involved in the pathophysiology of MDD. It would be desirable to simultaneously evaluate as many inflammatory markers as possible and correlate them with a comprehensive clinical assessment in order to understand the complex network of interactions between them (interactomic approach) [118,119].

It is noteworthy that some depressive patients may have elevated CRP and IL-6 levels, even in the absence of inflammation [44].

### 3.3. Tumor Necrosis Factor-α

#### 3.3.1. As a Diagnostic Biomarker

Tumor Necrosis Factor-α is another inflammatory cytokine, constantly and repeatedly reported to be increased in depression as compared to healthy controls [22,40,53,87,90,96,98]; however certain meta-analyses found this to be inconclusive [42,120]. Moreover, number of depressive episodes is associated with increased TNF-α concentrations [121].

#### 3.3.2. As a Diagnostic Biomarker

Baseline levels of TNF-α are not associated with a subsequent response to treatment [115]. Antidepressant treatment significantly decreases TNF-α concentrations, but only in responders. Persistently elevated levels of TNF-α during the course of treatment are associated with poorer outcomes, thus identifying patients with TRD [96,122]. Similar effects have been reported for electroconvulsive therapy (ECT)—clinical improvement following electroconvulsive therapy (ECT) correlated with a decline in TNF-α concentrations [123]. Hence, TNF-α could be a valuable marker of treatment resistance (a treatment-response marker) and a potential new biological target for the pharmacotherapy of depression. Anti-TNF-α drugs (infliximab, etanercept) have previously been reported to attenuate depressive symptoms [117].

#### 3.3.3. TNF Receptors

The inflammatory effect of TNF-α is mediated by its receptors—TNF-R1 and TNF-R2, both of which are expressed on cell surfaces. However, under certain conditions, they can be released into plasma. Once released, they are no longer active and act as ‘decoy’ receptors, binding circulating TNF-α molecules and thus regulating TNF-α signaling [124]. Depression is characterized by elevated serum TNF receptor levels, which are regarded as state [78] or trait [125] biomarkers.

Interestingly, Jannelidze et al. [126] demonstrated that IL-6 and TNF-α are elevated in patients at an increased risk of suicide, indicating that they may be regarded as state biomarkers. It has also been proven that the two cytokines directly inhibit adult hippocampal neurogenesis [127].

### 3.4. Interleukin-1

IL-1β is one of the major pro-inflammatory cytokines and together with TNF-α and a few other cytokines is thought to be responsible for the occurrence of sickness behaviour. A central or peripheral injection of IL-1β produces such a behaviour in mice. Interestingly, a similar effect has been described for lipopolysaccharide (LPS, endotoxin)—an injection of endotoxin leads to ventral striatum deactivation, diminished reward processing and anhedonia [128,129,130]. An increase in IL-1β in depression remains controversial. Certain meta-analyses support it [46,120] while others do not indicate such an association, including a recent umbrella review conducted by Carvalho et al. [89,90]. Such discrepancies may be caused by increased body mass index (BMI), which contributes to an increase in IL-1β [89], or the fact that IL-1 concentrations increase with the number of depressive episodes [121]. Conversely, Talarowska et al. [131] did not find significant differences in IL-1 concentrations between patients suffering from their first or successive depressive episodes. IL-1 also holds promise as a potential prognostic biomarker since elevated levels of IL-1β mRNA in blood can predict a poorer response to antidepressant treatment [132].

IL-1, TNF-α and IL-6 exert their pro-depressive effects by inhibiting neurogenesis [127,128], inducing apoptosis [129,130], negatively affecting synaptogenesis, synaptic plasticity and connectivity as well as the structure of synaptic membranes [131,132].

### 3.5. Interleukin-1 Receptor Antagonist

IL-1 receptor antagonist (IL-1RA) is a protein which competes with IL-1, binding to IL-1 receptor. Its production is stimulated by pro-inflammatory agents (e.g., IL-6) and therefore, elevated levels of IL-1RA indicate an enhanced inflammatory response. However, IL-1RA itself actually inhibits IL-1β and IL-1α signaling and as such is part of CIRS [35,79]. Some studies have reported that IL-1RA is elevated in depression [90,98], but no such association was found in a recent umbrella review by Carvalho [40]. Soluble IL-1RA is elevated both in unipolar depression and BPD, which excludes it as a marker for differentiating between these two disorders. It remains elevated even in affective remission and therefore it may be perceived as a trait biomarker for depression [125]. Sowa-Kućma et al. [100] demonstrated that sIL-1RA was positively correlated with a number of hospitalizations due to depression within a year, before being tested in affected individuals.

### 3.6. Interleukin-2, Soluble Interleukin-2 Receptor

IL-2 is a key cytokine in T lymphocytes activity [133,134], the function of which is disturbed in depression [135]. The effects of IL-2 are mediated by IL-2 receptor (IL-2R), present on cell membranes of activated T cells. IL-2R may also be cleaved into the bloodstream. It has been reported by a number of authors that soluble IL-2R (sIL-2R) is elevated in the blood of patients with depression and BPD [40,87,90,98]. Plasma sIL-2R could serve as a surrogate marker of T lymphocyte activation and IL-2 production [78]. sIL-2R may have immune-suppressant, immune-regulatory or immune-stimulatory properties, although most studies describes it as immune-suppressant [136].

### 3.7. Interleukin-10

Interleukin-10 (IL-10) is one of the most important anti-inflammatory cytokines. Elevated IL-10 concentrations in blood, along with enhanced IL-4 levels, may play a role in regulating IRS. In a meta-analysis performed by Kohler et al. [90], elevation of IL-10 was associated with depression. Two other meta-analyses found that IL-10 concentrations decrease with antidepressant treatment, making it a promising marker of treatment efficacy [83,88]. No differences in IL-10 concentrations between the first and subsequent depressive episodes were noted [137].

### 3.8. Interleukin-8

Data regarding IL-8 concentrations in depression are inconsistent. It has been reported that IL-8 is elevated in severe depression and positively correlated with depressive symptoms [138], which could suggest its role as a state marker. However, a meta-analysis by Eyre et al. [139] did not establish significant differences in IL-8 concentrations between patients with MDD and healthy controls. Lower baseline IL-8 levels were associated with a better response to antidepressant treatment in a recent meta-analysis [122], thus supporting its role as a prognostic biomarker.

### 3.9. Interleukin-4

Interleukin-4 (IL-4), one of the most important anti-inflammatory cytokines, was recently found to be downregulated in depression in a meta-analysis by Osimo [22]. IL-4 is produced by Th2 lymphocytes. IL-4 increases the production of anti-inflammatory sIL-1RA and inhibits M1 macrophage polarization, thus decreasing the release of IL-1β, IL-6 and TNF-α, as such contributing to CIRS [78].

### 3.10. Interferon-γ

INF-γ is a pro-inflammatory cytokine produced by Th1 lymphocytes. It is indicated that it causes microglial activation (shift) which contributes to depression [140]. Udina et al. [141] reported a higher risk of depression in patients treated with interferon for hepatitis C. As many as 40% of cancer or hepatitis C patients treated with INF-γ develop depressive symptoms and present with increased IL-6 levels. In depressed patients undergoing INF-γ therapy, lower tryptophan (TRP) and serotonin (5-HT) levels and higher kynurenine levels were noted in the peripheral blood [142,143,144]. Interestingly, the Combining Medications to Enhance Depression Outcomes (CO-MED) trial demonstrated a decrease in INF-γ after antidepressant treatment, which correlated with a lack of remission [145].

### 3.11. Macrophage Migration Inhibitory Factor

Macrophage migration inhibitory factor (MIF) is a pro-inflammatory cytokine that plays a role in hippocampal neurogenesis in animal models [146]. Decreased or elevated MIF levels have been reported in patients with MDD [147,148].

### 3.12. Transforming Growth Factor-β

Transforming growth factor-β (TGF-β) was analyzed in several studies as a potential marker for depression. However, recent meta-analyses did not prove significant changes in TGF-β levels in patients with MDD [40,90].

### 3.13. Other Cytokines

There are single reports in the available meta-analyses concerning increased concentrations of other cytokines—interleukin-3 (IL-3), interleukin-12 (IL-12), interleukin 13 (IL-13) and interleukin 18 (IL-18) [22,90]. However, their exact role and importance remains to be elucidated.

In a meta-analysis performed by Osimo et al. [22], three biomarkers, CRP, IL-12 and sIL-2R, presented significantly lower variability in patients with MDD compared to healthy controls.

### 3.14. Chemokines

Monocyte Chemoattractant Protein-1/Chemokine ligand 2 (MCP-1/CCL2) is responsible for the chemoattraction of monocytes, dendritic cells and memory T cells to the site of inflammation. Higher concentrations of this chemokine were found in depressed patients in comparison to healthy controls in a meta-analysis [139].

Furthermore, higher levels of eotaxin prior to antidepressant treatment, compared to its concentration following treatment, correlated with clinical remission [145].

### 3.15. Complement Proteins C2 and C3

The activity of complement is disturbed in depression. Levels of acute phase protein complement C3 are elevated in depression and are significantly higher in the atypical subtype compared to melancholic depression [149,150,151].

### 3.16. Bone Inflammatory Markers

Depressive patients have decreased bone mineral density and thus altered levels of bone inflammatory markers—osteo-protegerin (OPG)-RANK-RANKL system and osteo-pontin (OPN). Ketamine—a recently discovered fast-acting antidepressant agent—corrects these abnormalities [152].

### 3.17. Acute Phase Proteins

Depression has been associated with disturbances in acute phase proteins (APP): ceruloplasmin, inter-alpha-trypsin inhibitor heavy chain H4 and complement component—1qC [153]. Haptoglobin has also been reported to be elevated in depression and to differentiate between depressive subtypes [14]. Depression has also been associated with increased plasma B2-microglobulin [154].

### 3.18. Erythrocyte Sedimentation Rate

Erythrocyte Sedimentation Rate (ESR) is a non-specific measure of inflammation. In rheumatoid arthritis (RA) patients who were also diagnosed with depression, higher ESR levels were observed compared to non-depressive RA patients [155].

There is substantial heterogeneity in results in depression–inflammation studies. Even meta-analyses reveal contradictory results. Hiles et al. [156] searched for sources of inconsistency in depression–inflammation studies. The main confounding issues identified were the following: accuracy of MDD diagnosis, BMI and comorbid illnesses. A meta-analysis by Kohler et al. [90] added age and smoking status to the list.

### 3.19. Neopterin

Neopterin, a marker of cellular immune system activation, was reported to be upregulated in depression in some studies, particularly in the melancholic subtype. Neopterin also allows for estimation of the extent of oxidative stress (its concentration rises along with an increase in ROS levels) and the extent of Th1 lymphocytes activation [23]. A positive response to ECT is associated with a decrease in neopterin levels in responders [157] and a reduction in the neopterin:biopterin ratio [158]. Every subsequent episode of depression is associated with a more substantial increase in the level of neopterin, serving as an episode number marker [159].

The summary of the results of meta-analyses investigating inflammatory markers observed in depression is presented in Table 2.

## 4. Oxidative&Nitrosative Stress Findings in Depression

The brain is particularly vulnerable to oxidative and nitrosative stress (O&NS) [160]. The hippocampus, cerebellar granule cells and amygdala are brain parts most susceptible to oxidative damage [161]. Psychological stressors induce cytokine production and an inflammatory response [162] which facilitates the generation of reactive oxygen and nitrogen species (ROS/RNS), leading to a pro-oxidant state. Clinical depression is accompanied by increased O&NS and impaired antioxidant status (e.g., lower TRP, tyrosine, albumin, zinc) [163]. Oxidative and nitrosative stress manifests itself, inter alia, by higher levels of mitochondrial reactive oxygen species, lipid peroxidation products, DNA and protein damage products [10]. Elevated levels of protein carbonyls reflect protein damage—8-hydroxy-2-deoxiguanosine (8-OHdG) and 8-oxo-7,8-dihydroguanosine (8-oxo-Gua) are markers of DNA and RNA damage, respectively while malonylo-dialdehyde (MDA) and F2-isoprostanes emerge as the effect of lipid peroxidation [164].

Oxidative and nitrosative stress products have been detected in peripheral blood, red blood cells (RBC), mononuclear cells, urine, CSF and postmortem brain tissue of depressed patients. It is not clear if O&NS in depression originates from the peripheral or central nervous system. There are, however, some indicators which could suggest its peripheral origin [163]. In rats with genetic susceptibility to depression, higher depression rates were observed after exposure to oxidative stress [165].

Antidepressant drugs have antioxidant properties which are thought to account for, at least partially, their antidepressant efficacy [166]. Interestingly, antioxidants are also described as having antidepressant properties [167].

Reactive oxygen species (ROS) are mainly generated in mitochondria as a side product of the respiratory chain. They destroy defense systems when overproduced, creating a vicious circle and enabling further ROS generation [168]. It is not clear how exactly ROS exert their detrimental impact on the brain. ROS overproduction is known to trigger pathological cascades, eventually leading to increased permeability of the blood-brain barrier, morphological changes in the brain and neuroinflammation [169]. Under pathological conditions, oxidative stress could also induce neurodegeneration via different mechanisms such as apoptosis, axonal damage and excitotoxicity [170].

Oxidative stress activates inflammatory pathways, as extensively reviewed by Moylan et al. [163], whereas inflammation increases oxidative stress (e.g., an increase in the levels of cytokines IL-1 and IL-6 leads to decreased levels of albumin, zinc and high-density lipoprotein—HDL) [171,172,173,174]. Among oxidative stress markers, lipid peroxidation markers appear to have the greatest potential in depression.

### 4.1. Total Antioxidant Capacity

Total antioxidant capacity (TAC) was found to be decreased in patients with MDD [164] and inversely correlated with severity of depression [175]. TAC did not increase in response to treatment or in remission [176], which suggests that TAC could be a trait biomarker for depression.

### 4.2. Peroxide

Elevated peroxide levels were confirmed in the sera of patients with MDD in a meta-analysis [164]. Maes et al. [177] found that peroxide levels are higher in the acute phase of MDD, but normalize if depression is long-lasting, which makes them potential state markers for MDD.

### 4.3. Nitric Oxide

L-arginine–NO pathways play a role in the pathophysiology of MDD and are altered by antidepressants [178,179]. The NO-producing enzyme, nitric oxide synthase (NOS), has two isoforms: neuronal NOS (nNOS), mainly involved in neurotransmission, and cytokine-inducible NOS (iNOS), which plays an important role in inflammation [180]. NO levels are higher in patients with MDD [181] and were reported to characterize depressive patients after suicide attempt [182], which could make them state markers and enable early identification of patients at risk of suicide. NO is also reported to be involved in the pathogenesis of INF-γ-induced depression [183]. The disease is associated with lower NO metabolite levels and decreased platelet-endothelial NO activity [184]. However, a meta-analysis performed by Jimenez-Fernandez et al. [185] did not confirm significant differences in total nitrites in patients with MDD as compared to healthy controls. Nevertheless, another meta-analysis published by Liu et al. [164] documented decreased serum nitrate following antidepressant therapy.

### 4.4. Superoxide Dismutase

Superoxide dismutase (SOD) is one of the most important antioxidant enzymes. A number of investigators have reported that SOD is altered in depression, but the results are inconsistent. The majority of studies have found that SOD activity is increased in depression [186,187,188,189,190], but opposite results have also been reported [191,192,193]. A meta-analysis by Jimenes-Fernandes et al. [185] revealed higher SOD levels in patients with MDD in comparison to healthy controls. Increased SOD in depression probably reflects activated defense against ROS and RNS [168]. Plausible explanations for inconsistencies in the published results are severity, stage and duration of illness as well as a possible biphasic response in ROS production [177,191,194]. It is worth noting that greater reductions in SOD activity were observed in recurrent depression [191].

As for the effect of antidepressant treatment on SOD activity, the results are also inconsistent [23]. This may be due to methodological differences such as different drugs used, heterogeneity of the disease, differences in severity and the number of episodes.

### 4.5. Other Enzymes

Catalase (CAT) and myeloperoxidase (MPO) are antioxidant enzymes, the activity of which increases during depressive episodes [176,195,196]. An increase in antioxidant enzyme activity during acute depressive episode is possibly due to the activation of compensatory mechanisms in response to increased oxidative stress. Antioxidant enzyme activity normalizes following treatment and therefore they could serve as state markers of depression [181]. Nevertheless, in a meta-analysis performed by Jimenez-Fernandez [185], differences in CAT appeared nonsignificant. Lower paraoxonase (PON) activity, a potent antioxidant linked to HDL activity, was found in the sera of patients with MDD [164]. At the same time, significantly increased activity of pro-oxidative xanthine oxidase was observed in patients with MDD [192].

### 4.6. Lipid Peroxidation Markers

Lipid peroxidation is caused by the action of ROS/RNS on lipids (e.g., cell membrane lipids). Early-stage lipid peroxidation is reflected by higher lipid hydroperoxide levels, whereas late-stage lipid peroxidation is characterized by an increase in malonylo-dialdehyde (MDA), 4-hydroxy-2-nonenal (4-HNE) and F2-isoprostanes levels [197]. Lipid peroxidation is more pronounced in patients with MDD than in controls [181]. In a meta-analysis by Mazereeuw et al. [198], lipid peroxidation was correlated with depression severity. Peripheral lipid peroxidation markers are good surrogate markers for their central concentrations [199].

#### 4.6.1. F2-Isoprostanes

F2-isoprostanes are products of arachidonic acid peroxidation. They are chemically stable, which makes them good and reliable markers of lipid peroxidation [200,201]. Higher concentrations of F2-isoprostanes have been found in urine [202,203] and blood [204,205] of patients with MDD. Meta-analyses confirmed that F2-isprostanes are upregulated in MDD [164,206] and correlated with severity of depression. Lindqvist et al. [115] demonstrated that higher baseline levels of F2-isoprostanes correlate with poorer treatment outcomes.

#### 4.6.2. Malonylo-Dialdehyde, Thiobarbituric Acid Reactive Substances

MDA upregulation in MDD is widely documented [164,185,186,187,188,189,194]. Interestingly, MDA concentrations are higher in subsequent depressive episodes compared to the first episode of MDD [191,193].

Thiobarbituric acid reactive substances (TBARS) are a measure of oxidative tissue damage which could be used instead of MDA, albeit with low sensitivity and specificity. Elevated TBARS levels are reported to be trait markers of depression [125].

#### 4.6.3. Lipid Peroxidation Markers Following Treatment

According to a meta-analysis, antidepressant treatment leads to a decrease in lipid peroxidation markers [198]. The majority of studies and meta-analyses report decreased MDA concentrations following antidepressant treatment [164,185,187,188] which correlates with clinical improvement [186,194]. However, some studies did not establish a direct association between lipid peroxidation marker concentrations and clinical remission during antidepressant treatment, suggesting that these two parameters may be causally related but desynchronized [55,207]. By contrast, Chung et al. [202] found that F2-isoprostane levels increase after antidepressant treatment and this increase is correlated with alleviation of depressive symptoms.

The exact mechanism of how antidepressant treatment impacts inflammatory processes and oxidative stress is not fully understood. Following recovery, depressive patients often start looking after themselves, live healthier lives, eat more nutritious food, and exercise, and it cannot be ruled out that the observed reductions in oxidative stress and inflammatory parameters are epiphenomena of such lifestyle changes [23].

### 4.7. Neoepitopes

Oxidative and nitrosative stress causes brain, muscle and nerve injury, which eventually leads to the formation of new epitopes (neo-epitopes) that can induce immunological IgM/IgG response against them [208]. Antibodies against neo-epitopes have been detected in depression [177,209,210]. Higher concentrations of IgM against conjugated oleic, palmitic, myristic and azelaic acids, MDA, phosphatidyl inositol (Pi), NO-modified neo-epitopes, such as NO-tyrosine, NO-arginine, NO-TRP, NO-bovine serum antigen as well as IgG against oxidized low-density lipoprotein (oxLDL) have been described [177,209,210,211,212]. Interestingly, elevated levels of oxLDL antibodies are also found in cardiovascular diseases (e.g., atherosclerosis), autoimmunological diseases (e.g., lupus) and in diabetes [177], which could partly explain the high comorbidity between these diseases and could suggest their common pathophysiological background.

### 4.8. Nucleic Acids Damage

Oxidative stress can also cause DNA damage, which, combined with less efficient DNA repair, leads to increased DNA damage in depressed patients and contributes to mitochondrial dysfunction [9]. 8-OHdG is a reliable marker of DNA damage [200,213]. Increased blood and urine levels of 8-OHdG have been found in depressive patients [115,213,214,215]. A meta-analysis confirmed the upregulation of 8-OHdG in patients with MDD [206]. Elevated 8-OHdG levels correlate with MDD severity [215,216], and are higher in recurrent depression as compared to the first episode of the disease [215]. Enhanced 8-OHdG levels have been reported after SSRI treatment, but only in non-responders [115]. Jorgensen et al. [216] found that the RNA analogue of 8-OHdG—8-oxo-Gua—was elevated in the urine of depressive patients. However, they also reported significant increases in 8-oxo-Gua after ECT.

### 4.9. Glutathione

Findings relating to glutathione in depression are inconsistent. Its levels are lower in patients with MDD in most studies [181,190]. However, glutathione peroxidase (GPx) activity is reported to be increased [186], decreased [190,193] or not altered [187] in depressed patients compared to healthy control groups. Differences in GPx between depressed patients and healthy controls are nonsignificant according to the findings of a meta-analysis [185].

Plasma glutathione peroxidase activity decreased after antidepressant treatment [186]. Interestingly, an antioxidant agent—N-acetylo-cysteine (N-ACC)—could be useful in the treatment of depression [217,218]. N-ACC mimics GPx activity, which could exert an antidepressant effect [168]. The effectiveness of N-ACC may suggest the contribution of oxidative stress to treatment resistance in depression.

### 4.10. Uric Acid

Decreased levels of antioxidant uric acid have been reported in MDD in a meta-analysis. The concentrations of uric acid increased after antidepressant treatment [164,185].

### 4.11. Albumin

Hypoalbuminemia has been described in depression [13,164]. An increase in albumin levels following antidepressant therapy has been confirmed in a meta-analysis [164].

### 4.12. Coenzyme Q

Decreased levels of antioxidant coenzyme Q (CoQ) which induce impaired antioxidant protection and enhanced production of damaging TNF-α have been reported in depression [219]. Decreased CoQ is associated with chronic fatigue syndrome which is closely related to fatigue and somatic (F&S) symptoms of depression, described by Maes [219].

### 4.13. Zinc

Zinc levels have been reported to be reduced in depression and to increase after antidepressant therapy in meta-analyses [164,185]. The role of zinc in MDD was reviewed by Styczeń et al. [220]. Decreased concentrations of CoQ and zinc are both hallmarks of TRD [173].

### 4.14. Vitamin C

Vitamin C is known to have antioxidant properties. Antidepressant therapy increases vitamin C levels in patients with MDD [164]. That is why, vitamin C could potentially be considered as treatment-response biomarker of depression.

## 5. Neuroendocrine Findings in Depression

### 5.1. The Role of the Hypothalamus–Pituitary–Adrenal Axis

The influence of stress and the hypothalamus–pituitary–adrenals axis (HPA axis) activity on the pathophysiology of depression has been extensively studied since the 1960s. Stress axis disturbances are present in approximately 50%–70% of depressive patients [15]. Elevated concentrations of corticotropin-releasing hormone (CRH), adrenocorticotropic hormone (ACTH), antidiuretic hormone (ADH) and decreased dehydroepiandrosterone (DHEA) levels have been reported in MDD.

#### 5.1.1. Cortisol

**Figure 4:**
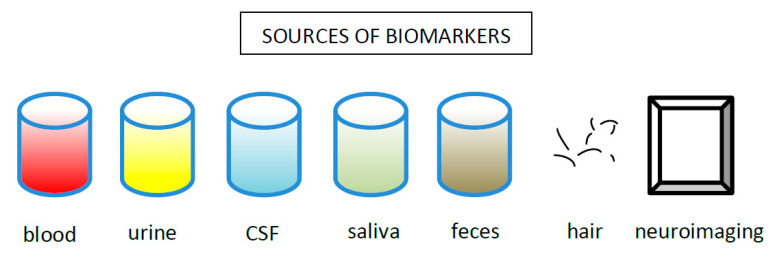
Sources of biological markers used in depression.

Chronic stress cause hypercortisolemia. Elevated cortisol levels have been repeatedly reported in depression [221,222]. Cortisol can be measured in different specimens such as blood, urine, saliva and even hair (Figure 4). By way of illustration, elevated cortisol in saliva after waking can serve as a biomarker for depression in adolescents [223] and an elevated cortisol concentration in hair could be a new measure of chronic stress exposure. An enhanced hair cortisol concentration could help differentiate between depression and other psychiatric disorders (Herane et al., 2015). Hypercortisolemia has been linked to severe cases of MDD, melancholic and psychotic depressive subtypes [21,224], psychogenic depression [225] and depression with ruminations [226]. A higher cortisol concentration predicts poorer outcomes of both psychological [227] and pharmacological treatment [228], and an elevated cortisol/DHEA ratio has been described as a marker for TRD, persisting after remission [229].

On the other hand, decreased cortisol concentrations are characteristics of atypical depression [224] and could be useful in differentiating between melancholic and atypical subtypes of the disease [230]. Hypo-cortisolism could also partly explain higher reward dependence and rejection sensitivity in patients with atypical depression [21].

Hypercortisolemia exerts a detrimental effect on the limbic system (particularly on CA3 neurons in the hippocampus). Under physiological conditions, the hippocampus and amygdala participate in feedback inhibition of the HPA axis through glucocorticoid receptors which are present in hippocampus cells [26]. The hippocampus damaged by elevated levels of cortisol is less efficient in HPA inhibition, which further enhances HPA hyperactivity, creating a vicious circle [11,221]. Cortisol-mediated decreased hippocampal cells proliferation and reduced neurogenesis lead to atrophic changes and volume reductions of the hippocampus which are observed in depressive patients [224,231,232,233].

It is not clear if the dysregulated HPA axis actually causes depression or if some other feature of depression is responsible for HPA malfunction. However, some depressive symptoms are undoubtedly produced by the dysfunctional HPA axis [11].

#### 5.1.2. Dexamethasone Suppression Test

In early studies, the dexamethasone suppression test (DST) was reported to identify melancholic depression [234,235]. However, later studies dampened enthusiasm and revealed its insufficient sensitivity [236] and specificity [237] to be a diagnostic biomarker. Nevertheless, DST could still potentially be used as a subtyping biomarker or state-dependent biomarker (as conversion from non-suppression to suppression in DST is correlated with a clinical response to antidepressant therapy) [238]. Moreover, an excessive cortisol response to the dexamethasone-suppressed CRH test (Dex-CRH test) after antidepressant treatment could predict a higher risk of recurrence [239].

#### 5.1.3. Corticotropin-Releasing Factor

The levels of corticotropin-releasing factor (CRF) are higher in some depressive patients [240]. However, in a quantitative summary, no significant increases in CRF in depressive patients compared to healthy controls were observed [222]. Interestingly, there are significant parallels between stress response, severe depression and central administration of CRF [240], which supports the involvement of CRF in the pathophysiology of depression. Higher levels of CRF mRNA and CRF have also been observed in the brain of depressive patients who committed suicide [241,242].

#### 5.1.4. Adrenocorticotropic Hormone

Elevated ACTH levels have been reported in patients with MDD [222]. Additionally, higher baseline ACTH levels in patients with BclI polymorphism in the glucocorticoid receptor gene predicted a poorer response to SSRI [243].

### 5.2. Thyroid Hormones

A significantly higher prevalence of thyroid dysfunctions is observed in patients with MDD/BPD [244]. Hypothyroidism may play a role in depressed mood [245], but the use of thyroid hormones as potential markers requires further investigation [180].

### 5.3. Nocturnal Melatonin Secretion

Melatonin, a derivate of serotonin, is a major hormone regulating the sleep-wake cycle. Diurnal melatonin secretion changes throughout the day. Nocturnal melatonin secretion has been reported to be higher [246] or lower [247] in patients with MDD in comparison to healthy controls. The phase angle between the cortisol acro-phase and dim-light melatonin onset has been proposed as a potential marker to distinguish individuals with MDD from healthy controls [248].

Interestingly, central administration of IL-1β decreased nocturnal melatonin secretion in sheep, which could suggest a link between inflammation, depression and sleep disturbances [249].

## 6. Growth Factor Findings in Depression

Growth factors are very promising markers for depression. Brain-derived neurotrophic factor (BDNF), vascular endothelial growth factor (VEGF), fibroblast growth factors (FGF) and VGF nerve growth factor are all involved in the pathophysiology of depression and are modulated by antidepressants. Moreover, they are present in the brain and in the periphery, which makes them suitable as biomarkers for psychiatric disorders [250,251,252,253]. Chronic stress impacts on the concentrations of growth factors. Reduced neurotrophic support inhibits neurogenesis (notably in the hippocampus and neocortex), which is likely to cause depression [11,254].

### 6.1. Brain-Derived Neurotrophic Factor

Brain-derived neurotrophic factor (BDNF) is by far the most extensively investigated growth factor in psychiatric research. Plasma BDNF can reflect central BDNF [255], which makes it a reliable peripheral biomarker of brain processes.

#### 6.1.1. Physiological Role

Under physiological conditions, BDNF plays a critical role in cellular resilience and neuroplasticity, enhances long-term potentiation (LTP) [11] and modulates the monoamine system. It also activates intracellular pathways such as mitogen-activated protein kinase/extracellular signal-regulated kinases (MAPK/ERK) pathways. Diminished MAPK/ERK pathway activity together with decreased cyclic adenosine monophosphate (cAMP) levels are known to be involved in the pathophysiology of depression [256].

#### 6.1.2. BDNF in Depression

Baseline BDNF levels are decreased in patients with MDD compared to healthy controls and the magnitude of a decrease in BDNF is negatively correlated with depression severity, as confirmed by meta-analyses [35,40,257,258,259,260]. Smoking [261] and diabetes [262] are accompanied by a decreased BDNF concentration in blood and both are independent risk factors for depression. Lower concentrations of peripheral BDNF mRNA in patients with MDD have also been found [94,263]. However, differences in BDNF mRNA expression were not related to symptom severity [263]. Two micro-RNA molecules—miR-132 and miR-182—regulate the expression of BDNF. Serum levels of these micro RNAs were significantly higher in unmedicated patients with MDD versus healthy controls, which was correlated with a decrease in serum BDNF levels [264].

Alterations in BDNF are not specific to MDD and can serve as a state biomarker in MDD, BPD and schizophrenia [265]. BDNF mediates the detrimental effect of HPA axis abnormalities on the brain [266]. Peripheral BDNF is neither a sufficient measure of MDD severity [260], nor does it discriminate between MB, BPD and schizophrenia. However, BDNF differentiates between mood states in BPD [267], and between acute and remitted states in MDD [265]. BDNF levels are also decreased in Alzheimer’s disease, which could support the hypothesis that depression belongs to the spectrum of neurodegenerative diseases [268].

The BDNF gene is induced by the cAMP response element binding protein (CREB) which binds to DNA sequences called CRE (cAMP response elements) and regulates BDNF gene transcription. The functions of CREB and BDNF are region-specific and vastly different in different brain parts. In the hippocampus they have an antidepressant effect, whereas in the ventral tegmental area and nucleus accumbens, BDNF produces a depression-like effect [11,224].

#### 6.1.3. BDNF as a Predictive Biomarker

Serum BDNF may act as a marker of predisposition to depression [269,270]. Decreased serum BDNF with normal cortisol levels may represent a relevant biomarker for individuals more likely to develop depression [180].

#### 6.1.4. Changes in BDNF Following Treatment

Decreased BDNF concentrations in depression normalize in response to pharmacological treatment [35,260,271,272,273] and ECT [274]. An increase in serum BDNF in response to antidepressant treatment successfully differentiates responders from non-responders [275]. However, antidepressant treatment causes an increase in BDNF levels even in the absence of clinical remission [260]. Therefore, BDNF has potential to be both a trait and a state biomarker [36].

The most widely used antidepressant drugs—SSRIs—produce an immediate increase in monoamine transmission but their mood-enhancing properties appear after weeks of treatment (Krishnan and Nestler, 2008a). The effect of antidepressant drugs is presumably mediated via changes in downstream events such as alterations in gene expression [276]. Apart from normalizing monoamine levels, antidepressants activate CREB which upregulates the expression of growth factors: BDNF, VEGF, VGF in the hippocampus. Growth factors promote hippocampal function, protect vulnerable neurons and, over time, lead to neurogenesis which eventually produces an antidepressant effect [224,232,277].

### 6.2. Insulin-Like Growth Factor-1, Growth Hormone

According to a recent umbrella meta-analysis, insulin-like growth factor-1 (IGF-1) is another growth factor which is significantly elevated in depression [40]. However, alterations in IGF-1 are not specific to MDD since IGF-1 is also enhanced in the manic phase of BPD [278]. There are promising preclinical studies in which the central or peripheral administration of IGF-1 increases hippocampal neurogenesis and decreases depressive symptomatology [279,280,281,282].

Decreased growth hormone (GH) levels have also been reported in patients with MDD [148,154].

### 6.3. Vascular Endothelial Growth Factor

VEGF is the main growth factor responsible for angiogenesis. Providing vascularization and blood support, it enhances neuron proliferation in the hippocampus [283]. VEGF may play a role in the pathogenesis of depression [250], although its exact role is not yet known. Data relating to VEGF levels in the blood of patients with MDD are not uniform, which may be due to the heterogeneity of depression and may reflect differences between its subtypes. However, the majority of studies indicate that VEGF is elevated in depression and normalizes under antidepressant treatment [284,285,286]. Higher VEGF concentrations have been observed in remitted MDD and in patients with a family history of depression [180].

### 6.4. Fibroblast Growth Factor-2

Significantly elevated FGF-2 levels have been reported in depressive patients [40,287]. Together with BDNF, FGF-2 is a second important growth factor marker in depression. Preclinical observations suggest that FGF-2 could mediate antidepressant effects [288]. Some connective tissue growth factors are co-activated in the inflammatory state and therefore their increase in depression may be the result of neuroinflammation [289].

### 6.5. VGF Nerve Growth Factor

VGF nerve growth factor concentrations have been found to be altered in depression, normalizing after antidepressant therapy, but only in clinical responders [94,290]. Additionally, it has potential as a treatment-response biomarker.

### 6.6. Nerve Growth Factor, Glial Cell Line Derived Neurotrophic Factor

The levels of nerve growth factor (NGF) and glial cell line derived neurotrophic factor (GDNF) are decreased in depression and the magnitude of dysregulation of these factors correlates with depressive symptoms severity. However, they do not change in response to antidepressant treatment [260].

## 7. Neurotransmitter Findings in Depression

Alterations in brain neurotransmitter levels—serotonin (5-HT), dopamine (DA) and norepinephrine (NA)—are considered a direct cause of depression. Even if this hypothesis is now thought to explain the pathogenesis of depression only partly, monoamine alterations in depression have been proven and the vast majority of antidepressant drugs currently used target monoamine systems. A search for monoamine-derived markers of depression is hindered by the fact that it is rarely possible to measure monoamine concentrations themselves. Scientists have to rely on peripheral monoamine metabolites, which do not necessarily reflect monoamine levels in the brain. Cerebrospinal fluid (CSF) content appears to reflect brain metabolites more accurately, but its acquisition is difficult and invasive. New imaging technologies are an invaluable tool for measuring brain neurotransmitters. However, a precise description of imaging biomarkers is beyond the scope of this review.

### 7.1. Serotonin

Serotonin is commonly known as a ‘happiness hormone’. Surprisingly, a decrease in 5-HT in the brain, measured by concentrations of serotonin metabolite 5-hydroxyindoleacetic acid (5-HIAA) in CSF, have not been found characteristic of depression itself, but rather of impulsivity [291], suicidality and a tendency to violence [292]. Serotonin exerts its action via the 5-HT1A receptor which has been reported to play a role in both prognosis and diagnosis of depression [293] as reduced 5-HT1A receptor binding is associated with depression [294]. Additionally, increased autoimmune responses to 5-HT were found to correlate with successive depressive episodes [295].

5-HT2A receptor can be found at blood platelets. The density of platelet 5-HT2A receptor tends to increase in patients with depression. However, it has been found to correlate more closely with suicidality than depression per se [296]. Increased 5-HT2A receptor density could potentially serve as a marker of suicide risk (state marker of depression).

### 7.2. Dopamine

Decreased dopamine levels in the striatum and cortex have been reported in depression [180]. Abnormalities in dopaminergic transmission have also been found in the nucleus accumbens and ventral tegmental, which are core parts of the brain reward circuit [297].

### 7.3. Noradrenaline

Noradrenaline is of major importance in MDD [298] and is perhaps the most promising source of neurotransmitter markers. There is a correlation between urine NA levels, and depression and anxiety symptoms [299]. Low urinary excretion of NA metabolite—3-methoxy-4-hydroxyphenylglycol (MHPG)—predicts a positive response to NA-selective drugs (e.g., imipramine) [300]. NA concentration in urine is a promising biomarker for guiding treatment selection and predicting its efficacy. MHPG together with a DA metabolite—homo-vanillic acid (HVA)—increase in line with a decrease in depressive symptoms. Lower levels of these metabolites predict a better response to SSRI.

### 7.4. Monoamine Oxidase Activity

Monoamine oxidases (MAO) are a family of monoamine-catabolizing enzymes. MAO-B is the most important of them. A reduction in MAO-B activity could be an early marker of response to antidepressant treatment [301].

### 7.5. Glutamate, GABA

Apart from the contribution of monoamines to the pathophysiology of MDD, dysfunction of glutamatergic transmission is also involved in the disorder [302]. Glutamate is a major excitatory neurotransmitter in the brain. Its upregulation causes excessive extra-synaptic N-methyl-D-aspartate receptor (NMDAR) activation leading to the influx of calcium ions (Ca^2+^) into neurons and accumulation of ROS in the neuron body [303]. As a consequence, it enhances the production of NO, which contributes to the occurrence of MDD [179,304].

Depression is associated with cortical hyper-glutamatergia and increased peripheral glutamate concentration [40,305,306]. Increased glutamate in MDD is closely related to decreased 5-HT and NA. Elevated levels of glutamate cause excitotoxicity, which contributes to the development of depression [180].

Gamma-aminobutyric acid (GABA) is known to be a major inhibitory neurotransmitter in the brain. Changes in glutamate and GABA increase the risk of oxidative stress and cell death [307]. Increased GABA enables the kindling action of glutamate and excessive glutamatergic activity leads to synaptic remodeling and neurodegeneration [180]. According to certain studies, an imbalance in the glutamate/GABA ratio could be a feature of depression [308]. GABA itself has been reported to be a trait biomarker for depression [309,310]. However, later studies revealed that it increases in response to antidepressant treatment [311].

## 8. Metabolic Findings in Depression, Lipidomics

To date, most studies of depression have focused on proteins. Phospholipids, however, account for 60% of dry mass of the brain [312] and play important biological roles, and hence particular emphasis should be placed on them in psychiatric research [313]. The lipid profile is disturbed in depression, but the exact character of the changes has not been fully elucidated [314]. Many lipid species have been linked to depression: glycerolipids, glycerophospholipids, sphingolipids, and triglycerides [315], and therefore it could be more effective to assess the entire lipid profile rather than particular types of lipid molecules separately. A higher BMI, which is frequently due to an excess of adipose tissue, is associated with a heightened risk of depression [316] and individuals suffering from depression are more likely to develop obesity [317]. It remains an open question as to whether lipid disturbances are a cause or consequence of depression. Among metabolic markers, polyunsaturated fatty acid (PUFA) disturbances and total cholesterol alterations appear to have the greatest potential as markers for depression.

### 8.1. Polyunsaturated Fatty Acids

A large number of lipidomic depression studies have investigated PUFAs. There are two main types of PUFA: omega-3 and omega-6. Both of them are present in the brain, but each one has a different mode of action. While omega-6-PUFAs (e.g., arachidonic acid) are pro-inflammatory, omega-3-PUFAs (e.g., eicosapentaenoic acid, EPA) possess anti-inflammatory properties [312,318]. Omega-3 acids increase the fluidity of cell membranes and exert a positive impact on neuronal development and neuronal transmission. In depression, blood levels of PUFAs are abnormal, with decreased eicosapentaenoic acid and other omega-3-PUFAs, and increased omega-6-PUFAs, including arachidonic acid concentrations, as reported in a number of studies [312,319,320,321]. Common depression comorbidities, such as cardiovascular diseases (CVD), diabetes, immunological and inflammatory activation, osteoporosis and cancer are also correlated with decreased omega-3-PUFAs [312]. A more significant decrease in omega-3-PUFAs is correlated with more severe depression [321]. Maes et al. [322] reported an elevated omega6/omega3 ratio in MDD. Elevated HDL and omega-3-PUFAs may have a protective effect on depression-mediated inflammation. Omega-3 supplementation (particularly EPA) improves treatment outcomes [323].

### 8.2. Cholesterol

Cholesterol also plays a role in depression. It does not cross the blood-brain barrier, but it is synthetized and recycled locally in the brain, mainly by oligodendrocytes [313]. A recent umbrella meta-analysis demonstrated that a decreased total cholesterol level is a highly suggestive marker for MDD [40,324]. As for cholesterol fractions, patients with MDD present with lower HDL and higher LDL concentrations, and a higher LDL/HDL ratio [164,325], which leads to immunological activation. Lower HDL may predict new-onset MDD in the older population [326].

### 8.3. Sphingomyelin

Sphingomyelin is a type of lipid found in the myelin sheath surrounding neural cell axons. The sphingomyelin 23:1 to sphingomyelin 16:0 ratio has been found to be inversely related to the severity of depression [327].

### 8.4. Adipokines

Patients with MDD have altered levels of adipokines [328]. Lower adiponectin levels have been reported solely in atypical depression [151].

### 8.5. Leptin and Ghrelin

Metabolic peptides—leptin and ghrelin—appear to be altered in depression, revealing a potential link between obesity and mood disturbances. However, the results of available studies are inconsistent—decreased, elevated or unchanged levels of leptin and ghrelin have been reported in depressive patients in comparison to healthy controls [329,330,331,332,333].

## 9. Proteomic Biomarkers

### 9.1. Insulin

Depression is frequently associated with impaired glucose tolerance, insulin resistance and diabetes [334,335,336]. Hyperglycemia contributes to inflammation in the brain which could cause depression.

According to certain studies, higher insulin levels in CSF might be the best biomarker to differentiate between patients with MDD and healthy controls [154].

### 9.2. p11 Protein

Another protein reported to be altered in depression is p11 protein. It is involved in serotonin signaling. Downregulation of p11 protein in NK cells and monocytes during antidepressant treatment correlates with a subsequent reduction in depression severity [337].

## 10. Transcriptomic Biomarkers

Transcriptomic biomarkers such as micro-RNA (mi-RNA) and long non-coding RNA (lncRNA) have also been investigated in depression. Pajer et al. [338] found a panel of 11 transcripts which were able to differentiate between the presence or absence of depression in animals and a panel of 18 transcripts common to depression and anxiety. Some of them may be more useful in diagnosing depression and some in predicting response to treatment [338,339]. Bocchio-Chiavetto et al. [340] demonstrated that 28 mi-RNAs are upregulated and 2 mi-RNAs are downregulated following antidepressant therapy.

It is worth noting that the increased expression of mRNA for pro-inflammatory cytokines IL-6, IL-1a, IL-1β, IL-8, IL-10, TNF-α, MIF, INF-γ) has been found in the peripheral blood of depressed patients [94,341,342,343].

More information on transcriptomic factors related to depression can be found elsewhere [36].

## 11. Kynurenine Pathway, Tryptophan

Tryptophan (TRP) is an amino-acid which is probably most directly implicated in the etiopathogenesis of depression. Under physiological conditions, it is transformed firstly into serotonin and then into melatonin. Serotonin is thought to regulate mood whereas melatonin is responsible for regulating sleep, both of which are disturbed in depression. Tryptophan depletion reduces 5-HT synthesis. The intensity of depressive symptoms correlates with the level of TRP depletion during antidepressant treatment [344].

Tryptophan is essential for T cell proliferation and cytotoxicity. Depletion of TRP (as is the case in depression) leads to T cell anergy [345] and subsequently to immunosuppression. A meta-analysis performed by Ogawa et al. [346] demonstrated reduced TRP levels in the plasma of patients with MDD. Decreased TRP could be a specific marker for MDD and BPD [180], and may play a central role in the pathophysiology of depression. It has been proven that injection of L-TRP modifies brain serotonin levels in rats [347]. On the other hand, injection of branched-chain amino-acids (valine, leucine, isoleucine), which compete with TRP, causes TRP and 5-HT depletion, and eventually, lowered mood [348]. A decrease in branched chain amino-acids following antidepressant treatment correlates with clinical improvement [349].

Apart from TRP depletion, an alternative pathway of TRP metabolism is activated in depression [350]. Systemic inflammation, with high levels of pro-inflammatory cytokines (e.g., INF-γ, TNF-α) along with elevated cortisol, produces sickness behaviour and facilitates the activation of IDO in the brain [345,351,352,353]. This enzyme transforms TRP into kynurenine [354] in the so-called TRYCATs pathway (tryptophan catabolites along the IDO pathway). Interestingly, the blockage of IDO reduces depressive symptoms without reducing sickness behaviour [352,353], which suggests that IDO is responsible for transformation from sickness behaviour to inflammation-induced depression. Enhanced activity of IDO has been observed in somatization, after suicide attempts and in adolescents with melancholic depression [23]. Interestingly, IDO also possesses antioxidant properties [355]. It has been demonstrated that alterations in the symptoms of depressed patients are positively correlated with kynurenine and negatively correlated with 5-HT concentrations [143]. The TRYCATs pathway produces kynurenine metabolites: 3-hydroxykynurenine (3-HK), 3-hydroxyanthranilic acid (3-HAA) and quinolinic acid (QA) which are cytotoxic and neurotoxic, affecting neurons and T lymphocytes [345]. QA acts as a NMDAR agonist, thus causing excitotoxicity. Its action could be reversed by ketamine—a NMDAR antagonist—recently described as a rapid-acting antidepressant. Enhanced levels of TRYCATs correlate with higher psychiatric rating scores in depressive patients [356], which could make them markers of depression severity. It should be noted here that most of the brain kynurenine originates from the periphery [345].

In terms of biochemical markers, the kynurenic pathway provides three highly suggestive markers for depression: decreased kynurenic acid (KYNA), decreased KYNA/3HK ratio and decreased KYNA/QA ratio [40,357]. Myint et al. [358] reported no changes in kynurenine pathway markers after antidepressant treatment.

An interesting distinction regarding the role of different types of glial cells in the kynurenic pathway has been made: KYNA—neuroprotective kynurenine metabolite—originates from astrocytes, while neurotoxic QA is produced only by microglia [359]. However, the exact role of astrocytes and microglia in depression is still to be elucidated.

## 12. The Role of Glial Cells

Glial cell disturbances contribute to the development of depression. It appears that there is a ‘creative balance’ between pro-inflammatory microglia, Th1 lymphocytes and M1 macrophages on one side and anti-inflammatory astroglia, Th2 cells, Tregs and M2 macrophages on the other. The former components are responsible for IL-1β, Il-2, IL-6, TNF-α, INF-γ production. The latter cells produce IL-4, IL-5 and IL-10. T cell activation with a Th1 shift is observed in depression [135]. Th1 cells activate IDO in the brain, which leads to neurotoxic QA synthesis. It activates NMDAR, which leads to hyper-glutamatergia and further Th1 activation.

In depression, the balance between glial cells is shifted towards microglial activation. Increased microglial activation and proliferation (MAP) is attributable to MDD, but not to BPD. Antidepressant treatment has been found to inhibit M1 microglia polarization [360]. On the other hand, astroglial loss in depression is reported in the anterior cingulate cortex, prefrontal cortex, amygdala and the white matter. The introduction of an astroglial-toxic agent—L-alpha-aminoadipic acid—provoked depressive symptoms in rats [361]. Glial loss leads to the release of cytokines which dysregulate glutamate metabolism leading to a further increase in cytokine concentrations. This leads to the upregulation of S100 calcium-binding protein B (S100B) and alterations in the blood-brain barrier function, which contributes to neuroinflammation. Elevated serum S100B levels have been observed during acute depressive episodes and mania [362]. Serum S100B has been found to correlate with suicidality in MDD and BPD.

## 13. Metabolomic Biomarkers

Depression research will undoubtedly take advantage of metabolomics—measuring small molecules (metabolites) in biological samples [363]. Scanning of the patient’s entire metabolome (a non-targeted approach) is a reasonable approach to identify new biomarkers and new pathways involved in depression. ELISA and Western-blot tests are then used to validate proposed biomarkers [6]. The metabolomic approach is a rapidly growing field with great potential for producing new biomarkers for depression.

### 13.1. Diagnostic Biomarkers

Metabolomic profiles are different in depressed individuals in comparison to healthy controls [364]. It has been demonstrated that a combination of plasma TRP, glutamate and cysteine can differentiate depressive patients from healthy controls [365]. Elevated plasma amino acid concentrations differentiated patients with melancholic depression from healthy controls [366]. In patients with MDD and heart failure, higher concentrations of amino-acids glutamate, aspartate and cysteine have been observed along with the dysfunction of fatty acids [367]. Downregulated N-methyl-nicotinamide and hippuric acid, and upregulated azelaic acid have been found in the urine of patients suffering from depression alone [368,369]. Paige et al. [364] found higher levels of lipid metabolites and neurotransmitter metabolites in the blood of elderly patients with MDD (dicarboxylic fatty acids, glutamate, and aspartate). GABA, citrate, glycerate, 9,12-octadecadienoate and glycerol concentrations were reduced in currently depressed patients [364]. A urinary biomarker panel for diagnosing patients with depression and anxiety was proposed by Chen et al. [370]. The simplified panel consisted of four metabolomic biomarkers: N-methyl-nicotinamide, amino-malonic acid, azelaic acid and hippuric acid. Significant differences in metabolic phenotypes between non-medicated depressed patients and healthy controls were revealed, whereas differences between non-medicated and medicated patients were found to be insignificant This may indicate that treatment of depression has a limited impact on metabolites in urine in the patient population [370].

A recently published systematic review performed by MacDonald et al. [371] analyzed metabolomic biomarkers for depression and BPD. The pathway that was most significantly affected both in MDD and BPD was the alanine, aspartate and glutamate pathway. For MDD and BPD, 10 out of 22 metabolic pathways were common. Those specific to MDD were valine, leucine, isoleucine biosynthesis and cyanoamino-acid metabolism [371]. Valine, leucine and isoleucine (branched-chain amino-acids) are involved in the formation of glutamate, which is a major excitatory neurotransmitter responsible for excitotoxicity [372].

In chromatography/nuclear magnetic resonance/mass spectrometry studies, the concentrations of eight metabolites appear to follow a specific trend (up-or downregulation) in urine, CSF and blood of depressed patients. These are increased glutamate, alanine, citrate, formate and decreased phenylalanine, valine, aminoethanol, and hippurate [371]. Glutamate, glycine and cysteine are required for the formation of glutathione [10]. Decreased GABA and increased lactate have been reported to be specific for MDD (MacDonald et al., 2019). The majority of key metabolites are involved in processes such as mitochondrial energy metabolism, signaling/neurotransmission and neuronal integrity [371].

In most studies using in vivo brain imaging techniques, a decrease in brain N-acetylaspartate (NAA), glutamate, creatine, GABA, GSH and phosphocreatine and an increase in brain choline and lactate have been observed [371]. Increased choline levels are in line with cholinergic hyperactivity and adrenergic hypoactivity, described in depression [373]. Mitochondrial dysfunction (e.g., due to oxidative stress) could cause anaerobic glycolysis which may explain elevated lactate levels in the brain. Aspartate is involved in the synthesis of glutamate and NAA. NAA is ubiquitous in neurons and is considered to be a marker of mitochondrial dysfunction and neuronal integrity [374]. NAA increases after antidepressant treatment, which further supports the neurotrophic effects of antidepressants [375].

Most robust biomarkers identified do not follow a specific up-or downregulation trend. This inconsistency is probably due to several variables which have not been taken into consideration in the review such as depressive subtypes, the patient’s age, sex, BMI, hormonal and smoking status [371]. Nevertheless, a diagnostic panel for MDD and BPD consisting of lactate, alanine, glycine, phenylalanine, tyrosine, sorbitol, pyroglutamate, aminoethanol and hippurate, and a panel for MDD alone comprising glutamate, citrate, valine and formate have been proposed [371]. It is worth noting that metabolomic research requires strict observance of the patient’s inclusion criteria and methodological procedures since the metabolome is highly variable and significant differences in results may appear.

### 13.2. Prognostic Biomarkers

Metabolomic markers may also potentially serve as prognostic markers in depression. Baseline levels of TRP, phenylalanine, purine and tocopherol could predict responders vs. non-responders to antidepressant treatment [376].

## 14. Intracellular Pathways

Intracellular signaling networks and transcription factors are likely to be dysfunctional in depression. The Janus kinases-signal transducer and activator of transcription (JAK-STAT) signaling pathway, glycogen synthase kinase-3 (GSK-3), and nuclear factors NF-κB and NRF-2 modulate inflammatory, O&NS and neuro-progressive pathways which are involved in depression [46]. By way of illustration, the expression of NRF-2 is regulated by oxidative stress and is altered in depression [10,377]. The expression of genes regulated by NRF-2 is upregulated in depression and downregulated after successful therapy [378]. The inhibitory impact of lithium on the GSK-3 pathway produces an anti-inflammatory effect and could partly explain the antidepressant effect of lithium [379].

Decreased adenosine triphosphate (ATP) levels have been found in post-mortem brains (dorsolateral prefrontal cortex) of depressed individuals [380]. Interestingly, ATP administration has been proven to have a fast antidepressant effect in mice [381].

A detailed review of intracellular pathway disturbances in depression is beyond the scope of this review and can be found elsewhere [382].

## 15. Genetics

Genetic contribution to MDD is around 40%–50% [383]. Several single nucleotide polymorphisms (SNPs) have been linked to depression, mainly those involved in monoaminergic and glutamatergic signaling [6]. Polymorphism in genes encoding the 5-HT transporter, 5-HT2A receptor, BDNF, TRP hydroxylase, SOD and CAT are candidate genes in the pathology of MDD [168,384,385]. Nevertheless, in an extensive Genome-Wide Association Study, no robust and meaningful genomic differences were found between MDD and healthy controls despite the large size of the study group [386]. A probable explanation may be the significant heterogeneity of depression and diverse or even opposite DSM criteria. Hence, a change in the paradigm may be necessary. Novel genomic approaches such as polygenic scores [387] or telomere length [388,389] could be more useful.

SNPs in several genes have been associated with response to antidepressant treatment [390], e.g., Met/Met genotype in the catechol-O-methyltransferase (COMT) gene [391]. However, no study has identified genetic variants that could be associated with treatment outcomes at a genome-wide statistical level [392].

## 16. Epigenetics

Epigenetic changes consist in modifications of gene expression without changes in the DNA sequence. They are mainly mediated by two processes: DNA methylation and histone modifications. Stress (both physical and psychological) is known to activate epigenetic mechanisms which increase the risk of depression [393]. Early life stress and polymorphism in the serotonin transporter gene facilitate methylation of the promoter region of the CRF gene in rats [394]. Stress during pregnancy leads to a higher risk of depression and anxiety in young adults. It is associated with reduced expression of the BDNF and AcH3K14 genes and increased expression of histone deacetylases in the hippocampus [395]. Elevated levels of methylation of the exon 1 promoter region in the BDNF gene have been found in patients with MDD in comparison to healthy controls [396].

## 17. Physiological Markers

Among ‘physiological markers’, alterations in circadian rhythms and electroencephalography (EEG) records have been observed in patients with MDD. Altered circadian rhythms are associated with genetic, environmental and developmental abnormalities preceding the development of MDD [397,398]. Korb et al. [399] reported that clinical response to anti-depressant treatment can be predicted by assessing activity in the rostral anterior cingulate cortex region in EEG.

## 18. Imipramine Binding

Imipramine was the first effective antidepressant drug in history. The binding of imipramine on the surface of platelets is considered a potential biological feature able to differentiate depressed individuals from healthy controls [400]. While several studies reported conflicting results, a meta-analysis performed by Ellis and Salmond [401] confirmed decreased maximal platelet imipramine binding (Bmax) in depressed patients. Nevertheless, the clinical utility of such a marker is questionable.

## 19. Treatment-Resistant Depression

A distinct problem in depression is the issue of treatment resistance. Early identification of patients at risk of treatment resistance may be possible with the use of biological markers. TRD has been associated with immune activation (enhanced mitogen-induced lymphocyte response, increased CD4/CD8 T cell ratio, enhanced IL-6 trans-signaling with higher sIL-6R, higher CRP and TNF-α), significantly enhanced oxidative stress (higher TBARS) and attenuated immune regulation (low sTNF-R2) in comparison to non-TRD patients [96,125,402,403]. IL-6, CRP, TNF and sTNF-R2 are associated with a number of failed antidepressant treatment attempts [404]. Risk factors for non-response also include elevated concentrations of circulating IL-1β, TNF-α, MIF and cortisol, dexamethasone non-suppression of cortisol, and decreased concentrations of IL-12, TSH, HDL, S100B, serotonin and noradrenaline [405].

## 20. Depression Subtypes

### 20.1. Melancholia

As mentioned above, depression is a heterogeneous disorder. This heterogeneity is an obstacle in biomarker research. In this section we would like to analyze in more detail two major depressive subtypes—melancholic and atypical depression.

The prevalence of typical and atypical depression in the general population is 7.1% and 3.5%, respectively [406]. Melancholic depression is a specifier of typical depression according to DSM-5. Approximately 20%–30% of all MDD cases are classified as melancholic depression [407,408].

The history of the term ‘melancholia’ is long and goes back to antiquity. Throughout the years, this type of depression has been described as endogenous, psychogenic, evolutionary, non-reactive, anhedonic, ‘vital depression type’ or depression with psychomotor retardation [407]. Melancholic depression is thought to be the most ‘pure’ endogenous depression. Even though it is difficult to establish precise boundaries of the term ‘melancholia’, a few characteristics are repeatedly reported in this subtype of depression. Motivational, appetitive and arousal functions are disturbed in melancholia (anhedonia, psychomotor retardation, hyperarousal, stress sensitivity) [407]. Melancholia is often characterized by greater severity, heritability, chronicity and a history of childhood trauma or abuse and comorbid anxiety [20,366,407,409,410]. Chronic low stress causes melancholic type behaviour in mice [411,412]. Melancholic depression is characterized by more significant psychomotor retardation and attention deficits in comparison to NMD [413]. Melancholic depression is more common in females and in advanced age, and is associated with greater severity and more common occurrence of psychotic features [414]. The strong heritable component in melancholia suggests that there may be an underlying biological dysfunction which could be manifested by certain biological features [366].

Melancholic depression appears to be associated with significant dysregulation of the DA system (e.g., higher frequency in people with Parkinson’s disease) [415]. Decreased dopaminergic function leads to psychomotor retardation which could underpin learned helplessness [416].

The presence of melancholic features is considered a risk factor for TRD [417]. In melancholic depression, response to SSRI treatment is poorer in comparison to response to drugs modulating multiple neurotransmitters (e.g., tricyclic antidepressants—TCA). This could be caused by a different circuitry characteristic of melancholia—more dopaminergic and noradrenergic, and not only serotoninergic [407,418,419]. In terms of treatment response, lower baseline plasma S100B protein may predict treatment resistance in patients with melancholic depression [420].

#### 20.1.1. Markers to Differentiate Melancholia from Healthy Controls

A significant number of studies have investigated biological features of melancholic depression. Among physiological markers of melancholia, lower systolic blood pressure, higher heart rate and a lower BMI have been found [230]. Melancholia is characterized by HPA axis hyperactivity, CRH dysfunction, higher plasma cortisol with altered cortisol diurnal variation, higher androstenedione and corticosterone (dysregulation promoting steroidogenesis in the upstream pathway), higher plasma arginine vasopressin, higher central NA, and basal hypothalamic-pituitary-thyroid ultra-sensitivity [230,366]. More pronounced inflammation or deficits in immune regulation have been reported in melancholic depression in comparison to healthy controls [230]. Upregulation of T cytotoxic CD8+ cells, M1 macrophages and Th1 lymphocytes [49,421] as well as downregulation of NK cells and Tregs have been found characteristic of melancholic depression. Melancholic depression is also characterized by increased IL-6 and sIL-6R (enhanced IL-6 trans-signaling) and decreased IL-1α and TGF-β [125,422].

Metabolomic biosignature differentiates patients with melancholic depression from healthy controls. Most metabolites related to lipids and metabolites related to stress hormone signaling are elevated in depression with melancholic features. One study demonstrated different levels of cystine, dodecanal, isoleucine, methionine, leucine, normetanephrine, and phenylalanine in melancholia [366]. Another study reported lower aspartic acid, glycine, GABA and higher NO levels in melancholic depression in comparison to healthy controls [423].

#### 20.1.2. Markers to Differentiate Melancholia from Atypical Depression

Since the performance of the pioneering research by Carroll et al. [235] in 1981, attempts have been made to differentiate two major depressive subtypes—melancholic and atypical—based on the biological profile. HPA hyperactivation and sustained cortisol elevation have repeatedly been indicated as distinct features of melancholic depression which can differentiate it from atypical depression [230,424,425]. Melancholic depression is characterized by a lower absolute monocyte count, increased haptoglobin, IL-6 and CRP, enhanced expression of T cell activation markers, and increased resistance of sIL-2R and IL-1β production in response to dexamethasone administration as compared to non-melancholic depression [426,427,428,429,430,431]. Higher triglycerides and fatty acids have been observed in the melancholic subtype [366]. Studies suggest that the angiotensin-converting enzyme (ACE) could be decreased in melancholic depression (vs. atypical, vs. healthy controls) [151,432], although published results are inconsistent.

Liu found decreased histamine and decreased arachidonic acid in melancholic depression [366]. The findings are in line with previous studies indicating that melancholic depression is characterized by immune repression in contrast to atypical depression which presents with inflammatory activation [222,424].

Among ‘physiological’ markers, differences in EEG patterns have been reported between melancholic and atypical depression [433].

### 20.2. Atypical Depression

Atypical depression differs more from healthy controls than melancholic depression. In a study by Lamers et al. [151], eight out of nine markers overlapped when the authors compared atypical depression with healthy controls and with melancholic depression. No marker reached statistical significance which would allow for differentiating between melancholic depression and healthy controls. The study demonstrated that in atypical depression the following molecules were altered as compared to melancholic depression: higher leptin, FABPa, complement C3, insulin, B2-microglobulin, ACE, and lower insulin-like growth factor-binding protein 1 and 2 (IGFBP1, IGFBP2) and mesothelin. When correcting for BMI, the effect remained significant only for IGFBP1, ACE and B2-microglobulin (the mediating effect of BMI) [151]. The results are not entirely consistent—enhanced leptin was also found in melancholic, but not atypical depression in a POWER (Premenopausal, Osteoporosis, Women, Alendronate, Depression) study [434].

In the majority of studies, atypical depression presents with a more disturbed metabolic profile. Atypical features correlate with a higher BMI, triglycerides and waist circumference, and lower HDL and obesity, which could partly explain elevations in IL-6, CRP, TNF-α, and IL-1β since adipose tissue enhances the production of pro-inflammatory agents [435]. However, metabolic disturbances cannot fully explain the pro-inflammatory shift characteristic of atypical depression [230].

Although some similarities were found between melancholic and atypical depression (e.g., elevated IL-6 and CRP concentrations) in a study by Lamers et al. [230], the authors suggest that inflammation per se is characteristic of atypical depression only, while melancholic depression is even thought to present an anti-inflammatory profile. Enhanced inflammation reported in the melancholic subtype in certain studies (e.g., elevated CRP, IL-6, TNF-α) could reflect the characteristics of the study cohort (e.g., patients with more severe symptoms of depression, inpatient). Decreased IL-4 and increased IL-2 have been reported in atypical as compared to melancholic depression [421].

## 21. Discussion

### 21.1. The Need for Markers

Psychiatric disorders, including depression, are still not completely understood. Knowledge regarding the etiopathogenesis of depression remains rudimentary. However, thanks to the implementation of new diagnostic techniques and technologies, particularly the ‘omics’ modalities, new evidence is emerging and our understanding of the complex nature of depression is becoming more profound. At present, depression is regarded as a disorder of communication between neurons, glia and endothelial cells, which is dependent on different systemic factors, including inflammation and oxidative stress [345]. However, this definition is probably incomplete. Biological markers constitute an invaluable aid in finding potential new patho-mechanisms involved in the pathogenesis of depression. Another important issue is a high level of treatment resistance in depression which occurs in more than one third of all MDD cases. Biological markers could help stratify patients into more homogenous subgroups and subtypes, such as melancholic and atypical depression, identify patients at risk of TRD or suicide and elucidate causes and mechanisms underlying these states, such as increased inflammation or pronounced oxidative stress. Biomarker levels can help assess the severity of depression, predict outcomes or guide adequate treatment selection. Lastly, by revealing underlying biological processes, biomarkers may help discover new drug targets and reduce the global depression burden.

### 21.2. Biomarkers That Have Potential

The selection of the most accurate biomarkers for depression is not an easy task. Research into depression markers has intensified in recent years, revealing a plethora of substances, gene polymorphisms, metabolites and other indicators of depression. Multiple meta-analyses present conflicting results, which may be due to a vast number of small, marginally significant studies, methodological differences between studies, depression comorbidities and the high heterogeneity of depression itself. Nevertheless, inflammatory biomarkers, biomarkers related to oxidative stress, HPA changes, growth factors and kynurenine pathway markers are repeatedly reported in depression studies and have promise to be reliable indicators of depression. The most recent and comprehensive, to date, umbrella meta-analysis by Carvalho et al. [40] demonstrates that depression is associated with increased CRP, IL-6, TNF-α, sIL-2R, IGF-1, FGF-2, glutamate and lipid peroxidation markers, and decreased BDNF, total cholesterol, KYNA, KYNA/3HK and KYNA/QA.

### 21.3. Looking for Biosignature

No individual marker for MDD has displayed sufficient specificity and sensitivity to be a diagnostic biomarker [436]. Absolute changes in HDRS—the most popular scale to measure depression intensity—are not significantly correlated with alterations in the levels of any particular biomarkers [115]. One of the biggest challenges (and opportunities) of current depression biomarker research is the inevitable and indispensable shift in the paradigm—from studies focused on one or two specific biomarkers (proteins, mutations, etc.) to a far more holistic approach, considering multiple biomarkers of different classes (i.e., a biosignature) [437] and interactions between them (interactome). It may also be the case that questionnaires used for the clinical assessment of depression are not best correlated with biomarker levels. Perhaps a more holistic approach to the patient’s state, including quality of life or everyday functioning, is needed to increase biomarker accuracy [438]. A possible lack of a link between various depression scales and depressive markers may also be due to high levels of comorbid anxiety in depressive patients since anxiety substantially impacts on the stress axis and hormones.

### 21.4. Issues

Biomarker research in psychiatry is particularly difficult due to a number of issues highlighted below.

#### 21.4.1. Lack of Specificity

Mental diseases have no sharp boundaries and there is a considerable overlap of symptoms between psychiatric entities. In addition, they could be perceived as spectrum disorders [439,440] and psychiatric biomarkers may have a transdiagnostic nature [441].

To address this issue, the Research Domain Criteria Initiative (RDoC) was launched by the National Institute of Mental Health. This postulates a totally new approach to the classification of mental disorders and seeks to connect observed behavioral dimensions with neurobiological systems [442]. It is designed in the form of a matrix concentrating on the assessment of different spheres of mental functioning affected by mental disorders (such as negative and positive valence systems, cognitive system, system for social processes and arousal/regulatory system) at different levels of analysis (genetic, molecular, cellular, neuroanatomical, behavioral, etc.) [33]. This approach goes across existing diagnostic entities, attempting not to replace the DSM-5 but rather to supplement it in a more biological, evidence-based way.

#### 21.4.2. Poor Understanding of the Biology of Depression

Another issue hindering depression research is a lack of broader understanding of its etiopathogenesis. This process has accelerated several times as a result of frequently accidental, discoveries of new antidepressant drugs. Studying the mechanism of action of these drugs eventually led scientists to the construction of new hypotheses of depression. That was the case with, inter alia, imipramine, initially designed as an anti-tuberculosis drug, which unraveled the monoamine mechanism of depression, and ketamine, an NMDAR antagonist, which underpinned the importance of NMDAR activation in the development of depression. However, our knowledge regarding the pathogenesis of depression is still rudimentary. Therefore, when it comes to studies of biomarkers, they frequently only correlate depression with altered levels of a few molecules, without providing a comprehensive explanation of the origin of the observed changes. Some studies ignore the fact that markers are interrelated in a complex, difficult-to-model network (e.g., some could be epiphenomena of others) [438]. Besides, the exact role of markers in healthy individuals and in depressive patients remains largely unknown.

To better understand depression, there is a need for reliable animal models. On the other hand, to build such models, a more comprehensive understanding of the pathophysiology of depression is necessary [11].

#### 21.4.3. Weak Studies

Recent technological advances have contributed to the intensification of research efforts in the field of biomarker discovery, particularly thanks to the expansion of ‘omics’ technologies which have revealed hundreds of putative biomarkers—gene polymorphisms, proteins and metabolites, whose presence (or altered levels—up or downregulation) could indicate depression. However, the published results are inconsistent and most markers lack robustness and validation and cannot be applied directly to clinical practice, which causes a ‘translational gap’ [6,40]. This is due to issues including a lack of clear definition of psychiatric illnesses, biomarker variability in individuals, widespread diffusion of small, underpowered studies characterized by ‘significance chasing’ and small effect size, ‘approximate replications’ of these studies which neither confirm decisively nor reject original findings (instead creating a penumbra of new hypotheses), selective publication of ‘positive’ results, selective reporting of outcomes, and finally, for most studies, focus on comparing ‘textbook patients’ with perfectly healthy individuals, which limits clinical application of such a biomarker [30,443]. A more profound understanding of individual variables such as patient’s age, sex, menstrual cycle, medication use, smoking status, BMI, and time of sample collection are important to ameliorate the accuracy of results [23].

#### 21.4.4. Heterogeneity

A search for biomarkers is also hindered by the heterogeneity of MDD [53,206,444]. A lack of robust, biologically validated, homogenous subgroups is one the greatest obstacles in establishing biomarkers for depression [3]. Making generalized statements about depression is difficult when one considers the plethora of different depressive subtypes described in the literature which are characterized by distinct symptomatologies. It might be worthwhile to use biological differences as a springboard for defining depression subtypes based on biomarker profile analysis utilizing latent class analysis [445].

#### 21.4.5. Brain-Periphery Differences

Peripheral fluids (blood, urine, saliva) are the most obvious sources of biomarkers in various diseases, including MDD. It remains an open question, however, to what degree biochemical changes in peripheral fluids reflect what happens in the brain/CSF. By way of illustration, even if cytokines can cross the blood-brain barrier and elevated levels of cytokines are present both peripherally and centrally in depressed patients [47,53,93,446], the peripheral cytokine profile should not be considered a simple reflection of what is happening in the brain since peripheral cytokines are strongly influenced by several extra-central nervous system (CNS) variables. A poor correlation between blood and brain biomarkers (with some overlaps) was described by Hayashi-Takagi et al. [447]. There are also discrepancies between studies caused by differences in investigated blood samples types (serum, plasma or cellular components).

To sum up, it is very unlikely that a single marker for MDD is established. However, even if the diagnosis of depression continues to be based on clinical signs, biomarkers may be a valuable tool for stratifying particular patients with the disorder, defining subtypes, improving treatment matching, avoiding specific treatment modalities, predicting response, etc. Such biomarker application is already common in other areas of medicine in diseases such as asthma, rheumatoid arthritis or cancer [30].

## 22. Limitations

The study is not systematic and does not provide quantitative information. The authors did not use strict inclusion and exclusion criteria. Both large and small studies were included. No age and gender bias were considered.

## Figures and Tables

**Table 1:** The main compounds of immune–inflammatory response system and compensatory immune–regulatory reflex system. Abbreviations: IL-1β—interleukin-1β; IL-2—interleukin-2; IL-4—interleukin-4; IL-6—interleukin-6; IL-10—interleukin-10; IL-1RA—interleukin-1 receptor antagonist; sIL-2R—soluble interleukin-2 receptor; sTNF-R1—soluble tumor necrosis factor receptor 1; sTNF-R2—soluble tumor necrosis factor receptor 1; TNF-α—tumor necrosis factor-α.

IRS	CIRS
IL-1β	sIL-1RA
TNF-α	sTNF-R1, sTNF-R2
IL-2 signaling	IL-2, sIL-2R
IL-6 trans-signaling	IL-6 classical signaling
Th1 and Th17 lymphocyte activation	Th2 lymphocyte activation with IL-4 production, Treg lymphocyte activation with IL-10 production
M1 macrophagic activation	

**Table 2:** Summary of inflammatory markers in depression according to meta-analyses performed throughout the years (left to right). Abbreviations: D—downregulated; U—upregulated;-—unchanged; CCL2—chemokine ligand 2; CRP—C-reactive protein; IL-1β—interleukin-1β; IL-3—interleukin-3; IL-4—interleukin-4; IL-6—interleukin-6; IL-10—interleukin-10; IL-12—interleukin-12; IL-13—interleukin-13; IL-18—interleukin-18; IL-1RA—interleukin-1 receptor antagonist.

	Howren 2009 [47]	Dowlati 2010 [53]	Liu 2012 [87]	Valkanova 2013 [89]	Haapakoski 2015 [42]	Strawbridge 2015 [96]	Goldsmith 2016 [98]	Kohler 2017 [90]	Ng 2018 [120]	Osimo 2020 [22]	Carvalho 2020 [40]
IL-6	U	U	U	U	U	U	U	U	U	U	U
CRP	U			U	U	U			-	U	U
TNF-α		U	U		-	U	U	U	-	U	U
sIL-2R			U				U	U		U	U
IL-1β	U	-			-			-	U		
IL-1RA							U	U			
IL-10		-						U			
IL-12								U		U	
IL-13								U			
IL-18								U		U	
TNF-R2								U			
CCL2								U			
IL-3										U	
IL-4		-						-		D	
INF-γ		-						D			
